# Prevalence and Antimicrobial Resistance of *Salmonella* Strains Isolated from Chicken Samples in Southern Italy

**DOI:** 10.3390/microorganisms13020270

**Published:** 2025-01-25

**Authors:** Pietro Di Taranto, Fiorenza Petruzzi, Giovanni Normanno, Carmine Pedarra, Gilda Occhiochiuso, Simona Faleo, Antonella Didonna, Domenico Galante, Lorenzo Pace, Valeria Rondinone, Carmelinda Trisolini, Laura Del Sambro, Matteo Beverelli, Roberta Catanzariti, Marta Caruso, Lucia Palazzo, Adriana Di Castri, Antonio Parisi

**Affiliations:** 1 Istituto Zooprofilattico Sperimentale della Puglia e della Basilicata, Via Manfredonia 20, 71121 Foggia, Italy; pietro.ditaranto@izspb.it (P.D.T.); domenico.galante@izspb.it (D.G.); laura.delsambro@izspb.it (L.D.S.); antonio.parisi@izspb.it (A.P.); 2 Dipartimento di Scienze Agrarie, Alimenti, Risorse Naturali e Ingegneria, Università degli Studi di Foggia, Via Napoli 25, 71121 Foggia, Italy

**Keywords:** *Salmonella* spp., chicken, serotyping, antimicrobial resistance

## Abstract

Salmonellosis is one of the most frequent foodborne zoonoses, and the consumption of contaminated poultry meat is considered the main source of *Salmonella* infections in humans. From 2021 to 2023, 384 chicken samples were collected from retailers in Apulia and Basilicata regions and analysed at the Istituto Zooprofilattico Sperimentale della Puglia e della Basilicata (IZSPB) laboratories. The *Salmonella* isolates were investigated to evaluate their phenotypic characteristics of antimicrobial resistance. A total of 125 (32.55%) samples tested positive for *Salmonella* spp. Three samples were simultaneously infected with strains of two different serotypes. Strains were classified into nine serotypes. Out of 128 strains, 112 (87.5%) isolates showed multidrug-resistant (MDR) profiles. Moreover, 16 isolates (12.5%) were ESBL producers with MDR profiles. Our data confirm that chicken products are a common source of *Salmonella* and highlight how *S*. Infantis was the most prevalent serotype (85.93%). Furthermore, *Salmonella* isolates showed antimicrobial resistance, which is very worrisome for animals and consumers. The high rate of antibiotic resistance along with the appearance of numerous MDR isolates is considered an actual concern and requires a robust surveillance network in a One Health concept.

## 1. Introduction

*Salmonella* sp. is a Gram-negative, non-spore-forming bacterium that belongs to the *Enterobacteriaceae* family. It is generally motile thanks to the presence of peritrichous flagella, except for *S*. Gallinarum and *S*. Pullorum that are non-flagellated [1]. *Salmonella* sp. is a ubiquitous enteric pathogen of wild and domestic animals. This plays an important role in the pathogen’s indirect or direct transmission to humans [2]. Symptoms of salmonellosis include gastroenteritis accompanied by fever, nausea, vomiting, abdominal pain, and diarrhoea. Headache and myalgia are common. The duration of fever and diarrhoea varies but is usually 2 to 7 days. Human salmonellosis is usually characterised by a self-limiting disease, depending on the host status. However, more severe symptoms, such as meningitis, osteomyelitis, endovascular infections, and septic arthritis, may occur in high-risk groups of people such as very young, elderly, and immunocompromised people [3].

Compared to other bacteria, *Salmonella* can survive for extended periods in dry environments and in water. It can be due to its ability to grow at temperatures between 5 °C and 47 °C with an optimal range from 32 °C to 35 °C in a pH from 4 to 9 with an optimal range from 6.5 to 7.5 [4]. A water activity (*a_w_*) of less than 0.94 can inhibit its growth [5].

*Salmonella* can be divided into two species: *Salmonella enterica* and *Salmonella bongori*. Furthermore, *S. enterica* has six subspecies: *enterica* (I), *salamae* (II), *arizonae* (IIIa), *diarizonae* (IIIb), *houtenae* (IV), and *indica* (VI) [6]. *Salmonella* serotyping is the traditional method used for the classification, characterisation, and surveillance of *Salmonella* worldwide. *Salmonella* serotyping is important in both human and veterinary diagnostics. Serotyping is based on the agglutination of somatic antigen (O antigen) and flagellar antigens (H antigens) with specific O and H antisera. According to the White–Kauffmann–Le Minor scheme, combinations of the O and H antigens can divide *Salmonella enterica* into more than 2600 serotypes, which are divided into 60 serogroups [7].

Regarding food safety, *Salmonella* sp. is one of the most common human foodborne pathogens in Europe [8]. The transmission of *Salmonella* typically occurs through the consumption of contaminated food or water [9]. In 2022, salmonellosis was the second most reported foodborne gastrointestinal infection in humans in Europe, with 1014 notified outbreaks, 6632 cases of illness, 1406 hospitalisations, and 8 deaths [10]. Italy was in sixth place among the European countries in terms of number of foodborne outbreaks, with 52 cases (5.12%), preceded by France (23.27%), Spain (22.18%), Poland (18.83%), Slovakia (11.14%), and Germany (6.8%). At the European level, ‘Meat products made from poultry meat intended to be eaten cooked’ was by far the matrix with the highest *Salmonella* prevalence (8.34%). Next came ‘fresh poultry meat’ and “Minced meat and meat preparations made from poultry meat intended to be eaten cooked”, which were the food categories with the second and third highest *Salmonella* prevalence (6.98% and 2.96%, respectively) [10].

A significant global risk to food safety and public health is represented by antimicrobial resistance (AMR) [11]. The widespread, prolonged, and inappropriate use of antimicrobials to enhance production to meet the rising demands for poultry meat has facilitated the increase in multidrug-resistant (MDR) foodborne pathogens, such as *Salmonella* spp. [12]. MDR *Salmonella* variants can cause severe illnesses, potentially leading to longer hospital stays [13]. Relevant studies show significant differences among *Salmonella* spp. MDR strains isolated from food and from animals suggest that food is the main source of MDR [2].

Starting from these assumptions, this study aimed to investigate the prevalence of *Salmonella* spp. isolated from official chicken food samples collected in Apulia and Basilicata regions from 2021 to 2023 during three years of official control activity as a part of the Italian Regional official control plans and the phenotypic characteristics of antibiotic resistance of the isolated strains.

## 2. Materials and Methods

### 2.1. Sample Collection

Overall, 384 chicken food samples were collected, which were distributed by the local veterinary authorities of Apulia and Basilicata regions between January 2021 and December 2023. In particular, 344 samples were collected in Apulia region, and 40 samples were collected in Basilicata region. The sampling was carried out according to the Multiannual Regional Control Plans (MRCPs) of Apulia and Basilicata regions. They were routinely analysed in the Istituto Zooprofilattico Sperimentale della Puglia e della Basilicata (IZSPB) laboratories as part of the official control activities of European regulations (Regulation (EC) no. 2073/2005) on microbiological food safety criteria [14]. They consisted of 320 fresh poultry meat samples (MEAT), 11 mechanically separated poultry meat (MSM) samples, 19 meat preparations made from poultry meat intended to be eaten cooked samples (PRP), 15 meat products made from poultry meat intended to be eaten cooked samples (PRD) and 19 ready-to-eat cooked chicken meal samples (RTE) (Table 1).

### 2.2. Salmonella Isolation and Serotyping

*Salmonella* strains were isolated from food samples according to ISO 6579-1:2017 + Amendment 1:2020 [15]. In the laboratories of the food microbiology unit of Foggia, *Salmonella* was identified to the genus level using MALDI-TOF MS (Matrix-Assisted Laser Desorption/Ionisation—Time of Flight Mass Spectrometry), following the producer guidelines (Bruker Daltonics GmbH, Bremen, Germany). *Salmonella* strains detected from samples analysed in the other IZSPB laboratories were confirmed thanks to the appropriate biochemical miniaturised tests (API 20E^®^, Biomerieux, Lyon, France) and serological tests. All *Salmonella* isolates were sent to the laboratories of the food microbiology unit of Foggia to be subjected to serotyping according to the White–Kaufmann–Le Minor scheme [7]. It was performed according to ISO/TR 6579-3:2014 using Statens Serum antisera (SSI Diagnostic, Hillerød, Denmark) [16]. One isolate per sample was stored at −20 °C using Microbank™ vials (Pro-Lab Diagnostics, Richmond Hill, ON, Canada).

### 2.3. Antimicrobial Susceptibility Testing

All confirmed *Salmonella* isolates were subjected to a phenotypic antimicrobial susceptibility test using the microbroth dilution method. It was performed with two antimicrobial panels (EUVSEC3^®^ and EUVSEC2^®^) used in the official antimicrobial resistance monitoring scheme in the EU (Commission Implementing Decision (EU) 2020/1729) [17].

Briefly, colonies were picked up from fresh culture grown overnight at 36 ± 1 °C on non-selective agar medium (Tryptic Soy Agar, VWR, Milan, Italy), and a McFarland standard 0.5 turbidity in sterile 0.9% NaCl solution was prepared. Then, 0.1 mL of each suspension was inoculated in 9.9 mL of cation-adjusted Mueller Hinton Broth (Becton Dickinson, Milan, Italy). All wells of the working plates (EUVSEC3^®^ plates, Termofisher Scientific, Paisley, UK) were filled with 50 µL of this suspension. Afterwards, the plates were incubated at 36 ± 1 °C for 24 h under aerobic conditions before the results were recorded. *Escherichia coli* ATCC^®^ 25922 was used for quality control as previously described.

The EUVSEC3^®^ plates contained a panel of 15 antibiotics. More specifically, ampicillin (1–32 g/mL) (AMP), azithromycin (2–64 g/mL) (AZY), amikacin (4–128 g/mL) (AMK), gentamicin (0.5–16 g/mL) (GEN), tigecycline (0.25–8 g/mL) (TGC), ceftazidime (0.25–8 g/mL) (TAZ), cefotaxime (0.25–4 g/mL) (FOT), colistin (1–16 g/mL) (COL), nalidixic acid (4–64 g/mL) (NAL), tetracycline (2–32 g/mL) (TET), trimethoprim (0.25–16 g/mL) (TRI), sulfamethoxazole (8–512 g/mL) (SUL), chloramphenicol (8–64 g/mL) (CHL), meropenem (0.03–16 g/mL) (MER), and ciprofloxacin (0.015–8 g/mL) (CIP).

*Salmonella* spp. isolates resistant to cephalosporine and carbapenems were then analysed to confirm extended-spectrum beta-lactamase (ESBL) production using the Sensititre EUVSEC2^®^ plate (Termofisher Scientific, Paisley, UK). The antibiotics on the EUVSEC2^®^ plate included the following: cefoxitin (0.5–64 g/mL), ertapenem (0.015–2 g/mL), imipenem (0.12–16 g/mL), cefotaxime (0.25–64 g/mL), ceftazidime (0.25–12/8 g/mL), cefepime (0.06–32 g/mL), cefotaxime/clavulanic acid (0.06/4–64/4 g/mL), ceftazidime/clavulanic acid (0.12/4–128/4 g/mL), and temocillin (0.5–128 g/mL).

The lowest concentration that inhibited the growth of bacteria was considered as the minimum inhibitory concentration (MIC).

ESBL production was confirmed based on the MIC results for cefotaxime, ceftazidime, cefotaxime/clavulanate, and ceftazidime/clavulanate. An ESBL producer was defined as an isolate showing at least a two- to three-fold decrease in concentration in its MIC for either cefotaxime or ceftazidime, tested in combination with clavulanic acid versus its MIC when tested alone.

Breakpoints used for the interpretation of the results were based on the epidemiological cut-off value (ECOFF), as shown in Report n. 14 Breakpoints EUCAST, except for azithromycin, chloramphenicol, nalidixic acid, tetracycline, and sulfamethoxazole, for which the sensibility or resistance values reported in the CLSI 2023 document M100 33rd edition were adopted [18,19].

Isolates that demonstrated resistance to three or more antimicrobials were categorised as multidrug-resistant (MDR).

## 3. Results

### 3.1. Prevalence and Serotype of Salmonella spp. Isolated from Samples

A total of 125 (32.55%) out of 384 samples tested positive for *Salmonella* spp. More specifically, 110 (34.37%) out of 320 fresh poultry meat samples, 5 (45.45%) out of 11 MSM samples, and 10 (52.63%) out of 19 meat preparations made from poultry meat intended to be eaten cooked samples tested positive. *Salmonella* spp. were not detected from PRD and RTE chicken samples (Table 2).

Out of the contaminated samples, 128 *Salmonella* strains were isolated because three samples were simultaneously infected with strains of two different serotypes. In particular, one fresh meat sample collected in 2021 was simultaneously infected with *S*. Infantis and *S*. Bredeney, one MSM sample collected in 2022 with *S*. Mdandaka and *S*. Enteritidis, and one fresh meat sample collected in 2023 with *S*. Infantis and *S*. Enteritidis.

*Salmonella* strains were classified into nine serotypes: *S*. Infantis (n = 110; 85.93%), *S*. Enteritidis (n = 6; 4.68%), *S*. Mbandaka (n = 3; 2.34%), *S*. Bredeney (n = 3; 2.34%), *S*. Kentucky (n = 2; 1.56%), *S*. Newport (n = 1; 0.78%), *S*. Agona (n = 1; 0.78%), *S*. Anatum (n = 1; 0.78%), and *S*. Thompson (n = 1; 0.78%) (Table 3).

### 3.2. Antimicrobial Susceptibility Testing

**Figure 1:**
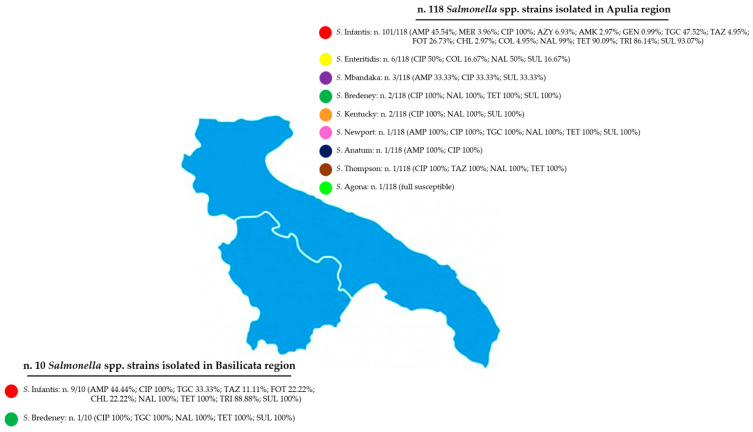
Map of the prevalence rate of AMR distribution in Apulia and Basilicata regions.

*Salmonella* isolates showed the following resistance percentages (Table 4): AMP (53/128) (41.41%), MER (4/128) (3.13%), CIP (122/128) (95.31%), AZY (7/128) (5.47%), AMK (3/128) (2.34%), GEN (1/128) (0.78%), TGC (53/128) (41.41%), TAZ (7/128) (5.47%), FOT (29/128) (22.66%), CHL (5/128) (3.91%), COL (6/128) (4.69%), NAL (119/128) (92.97%), TET (105/128) (82.03%), TRI (95/128) (74.22%), and SUL (111/128) (86.72%). Three isolates (2.34%) were sensitive to all antibiotics. The nine serotypes isolated with antimicrobial resistance are reported as follows (Table 5): *S*. Infantis (110/128) AMP (45.45%), MER (3.64%), CIP (100%), AZY (6.37%), AMK (2.73%), GEN (0.91%), TGC (46.36%), TAZ (5.45%), FOT (26.36%), CHL (4.55%), COL (4.55%), NAL (99.09%), TET (90.91%), TRI (86.36%), and SUL (93.64%); *S*. Enteritidis (6/128) CIP (50%), COL (16.67%), NAL (50%), and SUL (16.67%); *S*. Mbandaka (3/128) AMP (33.33%), CIP (33.33%), and SUL (33.33%); *S*. Bredeney (3/128) CIP (100%), TGC (33.33%), NAL (100%), TET (100%), and SUL (100%); *S*. Kentucky (2/128) CIP (100%), NAL (100%), and SUL (100%); *S*. Newport (1/128) AMP (100%), CIP (100%), TGC (100%), NAL (100%), TET (100%), and SUL (100%); *S*. Anatum (1/128) AMP (100%), and CIP (100%); *S*. Thompson (1/128) CIP (100%), TAZ (100%), NAL (100%), and TET (100%). These results were related to two regions in which samples were collected. In particular, 118 (92.18%) out of 128 strains were isolated in Apulia region as follows: *S*. Infantis (101/118) AMP (45.54%), MER (3.96%), CIP (100%), AZY (6.93%), AMK (2.97%), GEN (0.99%), TGC (47.52%), TAZ (4.95%), FOT (26.73%), CHL (2.97%), COL (4.95%), NAL (99%), TET (90.09%), TRI (86.14%), and SUL (93.07%); *S*. Enteritidis (6/118) CIP (50%), COL (16.67%), NAL (50%), and SUL (16.67%); *S*. Mbandaka (3/118) AMP (33.33%), CIP (33.33%), and SUL (33.33%); *S*. Bredeney (2/118) CIP (100%), NAL (100%), TET (100%), and SUL (100%); *S*. Kentucky (2/118) CIP (100%), NAL (100%), and SUL (100%); *S*. Newport (1/118) AMP (100%), CIP (100%), TGC (100%), NAL (100%), TET (100%), and SUL (100%); *S*. Anatum (1/118) AMP (100%) and CIP (100%); *S*. Thompson (1/118) CIP (100%), TAZ (100%), NAL (100%), and TET (100%). The 10 (7.82%) out of 128 strains isolated from samples collected in Basilicata region showed the following resistance: *S*. Infantis (9/10) AMP (44.44%), CIP (100%), TGC (33.33%), TAZ (11.11%), FOT (22.22%), CHL (22.22%), NAL (100%), TET (100%), TRI (88.88%), and SUL (100%); *S*. Bredeney (1/10) CIP (100%), TGC (100%), NAL (100%), TET (100%), and SUL (100%) (Figure 1). Out of 128 strains, 112 (87.5%) isolates showed MDR profiles (Table 6). Moreover, 16 isolates (12.5%) were confirmed to be ESBL producers.

## 4. Discussion

The present work reports the results of a three-year investigation related to the prevalence and phenotypic characteristics of antibiotic resistance of *Salmonella* spp. isolated from official chicken food samples collected in Apulia and Basilicata regions.

The obtained data report an overall prevalence of 35.55%. The highest prevalence was registered in 2022 (36.09%), with 48 positive samples out of 133, followed by the prevalence in 2023 (33.58%) and 2021 (27.35%), with 45 out of 134 and 32 out 117, respectively.

Our findings are higher than the results reported in a three-year survey conducted on fresh chicken meat samples marketed in Italy, with an overall prevalence of 7.49%. More specifically, 1.31% out of 306 samples tested positive in 2016, 7.89% out of 304 samples in 2018, and 14.4% out of 257 samples in 2020, respectively [20].

However, the data obtained in our work are similar to the results described in a study carried out from 2002 to 2013, where *Salmonella* was detected in 30.5% of chicken meat samples [21].

Our matrices with the highest *Salmonella* contamination were meat preparations made from chicken meat intended to be eaten cooked with 52.63%, followed by MSM samples (45.45%) and fresh chicken meats (34.37%). These values might reveal that the contamination can be due to the inadequate handling and inefficient hygiene practices during the slaughtering and preparation. This can lead to the spread of *Salmonella* in chicken meats, becoming a high-risk factor for foodborne outbreaks.

Serotyping on 128 *Salmonella* strains isolated from our samples highlights how *S*. Infantis was the most prevalent serovar (85.93%). Our finding was in line with data reported in the literature. Since 2014, *S*. Infantis has indeed become the primary serovar isolated in broiler production in Europe. Presently, broilers and their derived products account for 95% of *S*. Infantis isolates [22]. Furthermore, *S*. Infantis was the most detected serotype (50.1%) out of the 13.061 strains isolated from chicken samples in 2023 and was the fourth most frequent cause of human salmonellosis in Europe [10]. The increasing prevalence of *S*. Infantis in poultry meat could be a result of the implementation of vaccinations against *S*. Enteritidis and *S*. Typhimurium in recent years [23].

*S*. Enteritidis was exclusively detected in six out of 125 samples with a prevalence of 4.8%. Our result is lower than the prevalence registered in the EFSA and ECDC reports, where 71.7% of *S*. Enteritidis was firstly related to broiler flocks and meat. These data are important because this serotype, with 67.3% of cases, is in first place of the five serotypes most involved in human infections at the European level, followed by *S*. Typhimurium (13.1%), a monophasic variant of *S*. Typhimurium (1,4,[5],12:i:-) (4.3%), *S*. Infantis (2.3%), and *S*. Derby (0.89%) [10].

The detection of three *Salmonella* Mbandaka strains from our samples confirms the presence of this serovar in chicken products. Since September 2021, *Salmonella* Mbandaka ST413 was, indeed, linked to a multi-country outbreak that occurred in the EU/EEA, Israel, and the UK, possibly due to the consumption of contaminated chicken meat [24].

The existence of double infections in the same sample suggests that broilers can be either infected simultaneously during a brief period either through one or multiple sources (i.e., food, water, environment, etc.) or that meat can be contaminated along the different steps of the food chain (i.e., slaughtering, handling, etc.). The presence of *S*. Agona and *S*. Anatum may be due to cross contaminations that occur in the working environments, considering that these serovars are mostly prevalent in turkey meat chains [10].

Regarding the phenotypic characteristics of antibiotic resistance, in our study, 87.5% (112/128) of *Salmonella* spp. strains showed multidrug-resistant (MDR) profiles, as they were resistant to more than three classes of antimicrobials.

In the current study, high resistance of *Salmonella* spp. was attributed to fluoroquinolones (95.31% to ciprofloxacin and 92.97% to nalidixic acid) and to third-generation cephalosporins (22.66% to cefotaxime and 5.47% to ceftazimide). They are classified as highest priority critically important antimicrobials (hpCIAs) in human medicine and often constitute first-line treatment for invasive salmonellosis in humans [25]. Possible resistances to these hpCIAs could lead to therapy failure, with severe public health consequences [26]. Fluoroquinolone-resistant non-typhoidal *Salmonella* is a major global concern because of its resistance. Fluoroquinolone-resistant non-typhoidal *Salmonella* can share resistance genes with typhoidal *Salmonella*. This can increase the risk of resistance transfer, compromising the effectiveness of fluoroquinolone in treating typhoid fever [27].

Obtained data registered resistance to ampicillin (41.41%), sulfamethoxazole (86.72%), and tetracycline (82.03%). These antimicrobials are categorised as Category D, (“Prudence”, the lowest risk category) by the European Medicines Agency (EMA), and they are widely used in veterinary medicine for treating infections in production animals [28]. Our results are important because the World Health Organisation (WHO) categorises ampicillin as a ‘critically important antimicrobial’ (CIA) in human medicine, while sulfamethoxazole and tetracycline are categorised as ‘highly important antimicrobials’ (HIAs) in human medicine [26].

Interestingly, 41.41% of *Salmonella* spp. strains were resistant to tigecycline, a Category A (“Avoid”) antimicrobial [28]. Tigecycline is used for the treatment of serious infections in adults caused by MDR Gram-positive and Gram-negative bacteria but not in food-producing animals (FPAs). Tigecycline is structurally related to the tetracycline class of antibiotics that are widely used in FPA. Currently, it has been demonstrated that resistance to tigecycline is conferred by mobile plasmid-mediated transmissible genes which have been detected in humans, animals, and environmental ecosystems [29]. So, the excessive use of tetracycline antibiotics in FPA may contribute to the emergence of plasmid-mediated genes, with the potential for spread to human bacterial species. Moreover, by analysing our data, we referred to 53 *Salmonella* strains resistant to tigecycline; we can notice that the resistance has been constantly increased throughout the three-year period, with 6/53 in 2021, 8/53 in 2022, and 39/53 in 2023, respectively.

The colistin resistance of *Salmonella* spp. found in our study was 4.69%. This finding is more elevated than data (2.5%) registered in the EFSA and ECDC reports [26]. Out of 110 *S.* Infantis strains, five (4.55%) isolates were resistant to colistin. These data are higher than the ones found in a study conducted on 85 strains of *S*. Infantis isolated from broilers slaughtered in Italy in the period 2016–2019, with a prevalence of 3.5% [30]. Additionally, one of six *Salmonella* Enteritidis strains was resistant to colistin. Colistin is a polymyxin, and it belongs to Category B (“Restrict”). Its use in animals should be restricted to mitigate the risk to public health [28]. Although the use of colistin in humans ceased in the early 1990s due to nephrotoxicity and neurotoxicity, our data are worrisome because it is used as a last resort antibiotic against MDR Gram-negative pathogens. As *S.* Enteritidis is the serovar involved in 67.3% of human infections, the veterinary use of colistin may allow for the spread of colistin resistance acquisition to humans via food.

The low resistance to meropenem found in our study (3.13%) confirms that sporadic cases of carbapenemase-producing non-typhoidal serovars (NTSs) of *Salmonella enterica* have already been reported in food [31]. Considering that horizontal gene transfer together with co- and cross-selection mechanisms can be involved in the development of carbapenem resistance in NTSs, new ways of transmission, including the food chain, have to be studied.

*Salmonella* spp. isolates showed low resistance to aminoglycosides, Category C (“Caution”) (2.34% to amikacin ad 0.78% to gentamicin, respectively) [28]. This finding does not correspond to data reported in a study conducted on *Salmonella* strains isolated from chicken samples, where 38 aminoglycoside-resistant genes were detected [32].

*S*. Infantis isolates showed high resistance to ciprofloxacin (100%), nalidixic acid (99.09%), sulfamethoxazole (93.64%), and tetracycline (90.91%). This resistance pattern is typical of a major European clone of *S*. Infantis, which is prevalent among broilers [33]. The high resistance to tetracycline for *S*. Infantis found in our work is similar to what was reported in other studies carried out in Italy. In fact, Proietti et al. (2020), Peruzy et al. (2020), and Castello et al. (2023) reported 96.5%, 86%, and 72.5% resistance to this antimicrobial from poultry meats, respectively [34,35,36]. In 2006, the implementation of an EU regulation banned the use of specific antibiotics, such as tetracycline, of relevance to human health as animal growth promoters; the increase in isolates resistant to this drug may be due to the misuse of tetracyclines in veterinary medicine for the treatment of infections in poultry in recent years [37,38]. Referring to sulfonamides, the high resistance to trimethoprim (86.36%) and sulfamethoxazole (93.64%) is in line with those found by other Italian authors [34,35,36]. Our data are worrying because these antibiotics are used as second-line therapies in humans who fail to respond to the first-line antibiotics and in those with persistence of symptoms [39]. It is important to highlight that the resistance to ciprofloxacin (100%) and to nalidixic acid (99.09%) for *S*. Infantis isolated from our poultry meat samples are the highest reported in Southern Italy [34,35,36]. This is of particular concern since fluoroquinolones are the gold standard for treatment against invasive salmonellosis in humans [35].

Referring to AMR distribution, it is not possible to make an equal comparison between the two regions given the substantial numerical differences in the samples. Analysing our data reported in Figure 1, it is important to point out that *S*. Infantis strains isolated in Apulia region are more concerning due to their resistances to carbapenems (MER 3.96%) and to colistin (COL 4.95%).

Despite Regulation (EC) no. 2073/2005, there are only regulations for specific *Salmonella* serotypes in fresh poultry meat such as *S*. Enteritidis, *S*. Typhimurium, and its monophasic variant; this study highlights that minor *Salmonella* serotypes, such as *S*. Infantis, have concerning characteristics of antibiotic resistance that can be considered as a serious threat to human and animal health [14].

The high rate of antibiotic resistance along with the appearance of numerous MDR isolates is very worrying. Additionally, a non-typhoidal *Salmonella* fluoroquinolone-resistant strain has been recently categorised as a high-priority pathogen. It means that *Salmonella* strains show increasing trends in resistance and are difficult to treat [27].

## 5. Conclusions

In conclusion, the data presented here compile an overview of the prevalence and distribution of the serovars and the AMR characteristics of *Salmonella* strains isolated from chicken samples in Southern Italy. This study confirms that chicken products are a common source of *Salmonella*. The prevalence of *Salmonella* found in our study (35.55%) along with its potential to transmit infections that are difficult to treat represents a threat for human health. So, in order to reduce the risk of *Salmonella* spreading, there is the need to alert food business operators to adopt and follow good hygienic practices when producing and handling chicken meats. The competent authorities should implement official controls on primary production (the correct use of antimicrobials in poultry plants) and postprimary production (good hygienic practices during the slaughtering, cutting, handling, and selling of meats). Finally, consumers should be informed using simple tools (web pages, social media, etc.) about the spread of AMR bacteria via food in order to apply good hygienic practices in their own kitchen, thereby avoiding cross contamination between raw chicken meat and other foods by disinfecting food contact surfaces very carefully.

Moreover, data reported in our study highlight that 87.5% of the *Salmonella* strains isolated from chicken products showed MDR profiles. It is important to underscore the resistance to colistin reported in our study, which is higher than the one showed in other papers and to tigecycline that is increasing over the years. Furthermore, although 95.31% of the isolates were represented by minor *Salmonella* serotypes, they showed worrying resistance patterns which may cause severe invasive diseases, in which antimicrobial treatments are compromised.

This work has highlighted the importance of the collaboration between local authorities and IZSPB laboratories in the context of the official food control in order to guarantee food safety and to protect the consumer health. Despite the use of antimicrobials, it is steadily declining in poultry farms in recent years in Italy; our study suggest that AMR is still an actual concern and requires a robust AMR surveillance network in a One Health concept.

## Figures and Tables

**Table 1:** Number and types of samples collected and analysed from 2021 to 2023.

Year	MEAT	MSM	PRP	PRD	RTE	Total
2021	93	6	6	7	5	117
2022	115	5	5	5	3	133
2023	112	0	8	3	11	134
Total	320	11	19	15	19	384

**Table 2:** Number and types of samples positive for *Salmonella* spp. from 2021 to 2023.

Year	MEAT	MSM	PRP	PRD	RTE	Total
2021	28	1	3	0	0	32
2022	40	4	4	0	0	48
2023	42	0	3	0	0	45
Total	110	5	10	0	0	125

**Table 3:** Prevalence rates of the nine serotypes related to the overall samples and to the overall isolates.

Samples	*Salmonella* spp. Isolates		Infantis		Enteritidis		Mbandaka		Bredeney		Kentucky		Newport		Agona		Anatum		Thompson	
n.	n.	P. %	S. P. %	I. P. %	S. P. %	I. P. %	S. P. %	I. P. %	S. P. %	I. P. %	S. P. %	I. P. %	S. P. %	I. P. %	S. P. %	I. P. %	S. P. %	I. P. %	S. P. %	I. P. %
384	128	32.55	28.64	85.93	1.56	4.68	0.78	2.34	0.78	2.34	0.52	1.56	0.26	0.78	0.26	0.78	0.26	0.78	0.26	0.78

Legend: P. = prevalence; S.P. = prevalence related to overall samples; I.P. = prevalence related to overall isolates.

**Table 4:** Resistance to single antimicrobial agents in *Salmonella* spp. strains. In bold are the highest priority and critically important antimicrobials (hpCIAs).

Antimicrobials	No. of Resistant Strains	No. of Intermediate Resistant Strains
Ampicillin	53 (41.41%)	0 (0%)
Meropenem	4 (3.13%)	0 (0%)
**Ciprofloxacin**	122 (95.31%)	0 (0%)
**Azithromycin**	7 (5.47%)	0 (0%)
Amikacin	3 (2.34%)	0 (0%)
Gentamicin	1 (0.78%)	0 (0%)
Tigecycline	53 (41.41%)	0 (0%)
**Ceftazidime**	7 (5.47%)	0 (0%)
**Cefotaxime**	29 (22.66%)	0 (0%)
Chloramphenicol	5 (3.91%)	16 (12.5%)
**Colistin**	6 (4.69%)	0 (0%)
**Nalidixic Acid**	119 (92.97%)	0 (0%)
Tetracycline	105 (82.03%)	0 (0%)
Trimethoprim	95 (74.22%)	0 (0%)
Sulfamethoxazole	111 (86.72%)	0 (0%)

**Table 5:** Resistance to single antimicrobial agents in the nine serotypes. In bold are the highest priority and critically important antimicrobials (hpCIAs).

Antimicrobials	Infantis	Enteritidis	Mbandaka	Bredeney	Kentucky	Newport	Agona	Anatum	Thompson
Ampicillin	45.45%	0%	33.33%	0%	0%	100%	0%	100%	0%
Meropenem	3.64%	0%	0%	0%	0%	0%	0%	0%	0%
**Ciprofloxacin**	100%	50%	33.33%	100%	100%	100%	0%	100%	100%
**Azithromycin**	6.37%	0%	0%	0%	0%	0%	0%	0%	0%
Amikacin	2.73%	0%	0%	0%	0%	0%	0%	0%	0%
Gentamicin	0.91%	0%	0%	0%	0%	0%	0%	0%	0%
Tigecycline	46.36%	0%	0%	33.33%	0%	100%	0%	0%	0%
**Ceftazidime**	5.45%	0%	0%	0%	0%	0%	0%	0%	100%
**Cefotaxime**	26.36%	0%	0%	0%	0%	0%	0%	0%	0%
Chloramphenicol	4.55%	0%	0%	0%	0%	0%	0%	0%	0%
**Colistin**	4.55%	16.67%	0%	0%	0%	0%	0%	0%	0%
**Nalidixic Acid**	99.09%	50%	0%	100%	100%	100%	0%	0%	100%
Tetracycline	90.91%	0%	0%	100%	0%	100%	0%	0%	100%
Trimethoprim	86.36%	0%	0%	0%	0%	0%	0%	0%	0%
Sulfamethoxazole	93.64%	16.67%	33.33%	100%	100%	100%	0%	0%	0%

**Table 6:** Patterns of antimicrobial resistance displayed by *Salmonella* spp. isolates.

Multiple-Resistant Pattern	Serotype	Resistance Pattern	No. of Isolates (%)	
No antimicrobial (full susceptible)	Mbandaka	-	1	(0.78%)
	Enteritidis	-	1	(0.78%)
	Agona	-	1	(0.78%)
One type of antimicrobial	Enteritidis	COL	1	(0.78%)
	Enteritidis	SUL	1	(0.78%)
	Mbandaka	SUL	1	(0.78%)
Two types of antimicrobials	Infantis	CIP, NAL	5	(3.91%)
	Enteritidis	CIP, NAL	3	(2.34%)
	Anatum	AMP, CIP	1	(0.78%)
	Mbandaka	AMP, CIP	1	(0.78%)
Three types of antimicrobials	Kentucky	CIP, NAL, SUL	2	(1.56%)
	Infantis	CIP, NAL, SUL	1	(0.78%)
Four types of antimicrobials	Infantis	CIP, NAL, TET, SUL	5	(3.91%)
	Bredeney	CIP, NAL, TET, SUL	2	(1.56%)
	Infantis	AMP, CIP, NAL, TRI	1	(0.78%)
	Thompson	CIP, TAZ, NAL, TET	1	(0.78%)
	Infantis	CIP, NAL, TRI, SUL	1	(0.78%)
Five types of antimicrobials	Infantis	CIP, NAL, TET, TRI, SUL	21	(16.41%)
	Infantis	CIP, TIG, TET, TRI, SUL	1	(0.78%)
	Infantis	AMP, CIP, NAL, TET, SUL	1	(0.78%)
	Infantis	CIP, TIG, NAL, TET, SUL	2	(1.56%)
	Bredeney	CIP, TIG, NAL, TET, SUL	1	(0.78%)
Six types of antimicrobials	Infantis	CIP, TIG, NAL, TET, TRI, SUL	20	(15.63%)
	Infantis	AMP, CIP, NAL, TET, TRI, SUL	8	(6.25%)
	Infantis	AMP, CIP, TIG, NAL, TET, TRI	1	(0.78%)
	Newport	AMP, CIP, TIG, NAL, TET, SUL	1	(0.78%)
Seven types of antimicrobials	Infantis	AMP, CIP, FOT, NAL, TET, TRI, SUL	9	(7.03%)
	Infantis	AMP, CIP, AZI, NAL, TET, TRI, SUL	1	(0.78%)
	Infantis	CIP, AZI, TIG, NAL, TET, TRI, SUL	1	(0.78%)
	Infantis	CIP, TIG, TAZ, NAL, TET, TRI, SUL	1	(0.78%)
	Infantis	CIP, AZI, TIG, COL, NAL, TET, SUL	1	(0.78%)
	Infantis	AMP, CIP, TIG, CHL, NAL, TRI, SUL	1	(0.78%)
	Infantis	AMP, CIP, TIG, NAL, TET, TRI, SUL	4	(3.13%)
	Infantis	AMP, CIP, CHL, NAL, TET, TRI, SUL	1	(0.78%)
Eight types of antimicrobials	Infantis	AMP, CIP, TIG, FOT, NAL, TET, TRI, SUL	10	(7.81%)
	Infantis	AMP, CIP, FOT, COL, NAL, TET, TRI, SUL	1	(0.78%)
	Infantis	AMP, CIP, TAZ, FOT, NAL, TET, TRI, SUL	2	(1.56%)
	Infantis	AMP, MER, CIP, FOT, NAL, TET, TRI, SUL	1	(0.78%)
	Infantis	AMP, CIP, TIG, CHL, NAL, TET, TRI, SUL	1	(0.78%)
	Infantis	AMP, CIP, TIG, CHL, COL, NAL, TRI, SUL	1	(0.78%)
Nine types of antimicrobials	Infantis	AMP, CIP, TIG, TAZ, FOT, NAL, TET, TRI, SUL	2	(1.56%)
	Infantis	AMP, CIP, AZI, TAZ, FOT, NAL, TET, TRI, SUL	1	(0.78%)
	Infantis	AMP, MER, CIP, TIG, FOT, NAL, TET, TRI, SUL	1	(0.78%)
	Infantis	MER, CIP, AMI, TIG, COL, NAL, TET, TRI, SUL	1	(0.78%)
Ten types of antimicrobials	Infantis	AMP, CIP, AZI, AMI, TIG, FOT, NAL, TET, TRI, SUL	1	(0.78%)
	Infantis	AMP, CIP, AZI, TIG, FOT, COL, NAL, TET, TRI, SUL	1	(0.78%)
Twelve types of antimicrobials	Infantis	AMP, MER, CIP, AZI, AMI, GEN, TIG, CHL, NAL, TET, TRI, SUL	1	(0.78%)
	Total		128	(100.00%)

## Data Availability

The original contributions presented in this study are included in the article. Further inquiries can be directed to the corresponding authors.
